# Errata

**Published:** 1889-02

**Authors:** 


ERRATA.—In Dr. Bell's notice of the Surgical Bureau of the I. H. A., in our January issue, page 39, line 15 from bottom, for *tend* read *attend;* page 40, line 8 from bottom, for *get to know* read *yet know.*

